# Increases in Depression, Self‐Harm, and Suicide Among U.S. Adolescents After 2012 and Links to Technology Use: Possible Mechanisms

**DOI:** 10.1176/appi.prcp.20190015

**Published:** 2020-09-09

**Authors:** Jean M. Twenge

**Affiliations:** ^1^ Department of Psychology San Diego State University San Diego

**Keywords:** depression, self‐harm, suicide, digital media, social media

## Abstract

**Objective:**

Increases in depression among adolescents have been concurrent with increases in digital media use. In this article, recent trends in mental health among U.S. adolescents and young adults are discussed and theories about their possible connection with concurrent increases in digital media use are presented.

**Methods:**

Large studies of trends in mental health in the 2000s and 2010s are described and possible mechanisms for the trends are discussed based on existing literature.

**Results:**

After remaining stable during the early 2000s, the prevalence of mental health issues among U.S. adolescents and young adults began to rise in the early 2010s. These trends included sharp increases in depression, anxiety, loneliness, self‐harm, suicidal ideation, suicide attempts, and suicide, with increases more pronounced among girls and young women. There is a growing consensus that these trends may be connected to the rise in technology use. Increased digital media and smartphone use may influence mental health via several mechanisms, including displacement of time spent in in‐person social interactions, individually and across the generation, as adolescent cultural norms evolve; disruption of in‐person social interactions; interference with sleep time and quality; cyberbullying and toxic online environments; and online contagion and information about self‐harm.

**Conclusions:**

U.S. adolescents and young adults are in the midst of a mental health crisis, particularly among girls and young women. The rise of digital media may have played a role in this problem via several mechanisms.

## HIGHLIGHTS

- Depression, self‐harm, suicide, and unhappiness suddenly increased among adolescents after 2012, especially among girls and young women.
- Increases in depression among adolescents have been concurrent with increases in digital media use.
- Increased digital media and smartphone use may influence mental health via several mechanisms, including displacement and disruption of in‐person social interactions, interference with sleep, cyberbullying, and online information about self‐harm.

In the early 2010s, reports began to surface that more adolescents and young adults were seeking help for mental health issues (1, 2). This finding could have indicated greater comfort with seeking help, however, rather than a true increase in the prevalence of mental health issues (3). To determine whether the prevalence of mental health issues has actually risen in the population of U.S. adolescents as a whole, data from unscreened, representative samples—not just those who seek help—are necessary (4). Below, I describe studies documenting recent trends in such samples.

## Trends in Mental Health Among U.S. Adolescents

Large screening studies (most nationally representative) of U.S. samples of adolescents and young adults since 2010 have shown declines in happiness, life satisfaction, and flourishing (5, 6) and increases in loneliness (7), anxiety (5), depressive symptoms (5, 8, 9), major depressive episodes in the past year (10, 11), hospital admissions for self‐harm behaviors (nonsuicidal self‐injury) (12), suicidal ideation (13), self‐harm and suicide attempts via poisoning (14), suicide attempts (13, 15), self‐reported suicidal ideation (11), and in the suicide rate (9, 11, 16).

**FIGURE 1:**
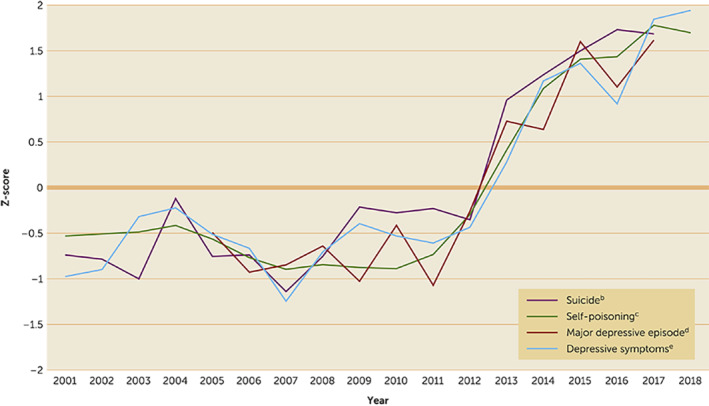
Indicators of poor mental health among U.S. girls and young women, 2001–2018^a^ ^a^Standard deviations are within means at the generational level, not at the individual level, and thus should not be used to calculate individual‐level effect sizes. ^b^Source: Centers for Disease Control and Prevention. Suicide rates among 12‐ to 14‐year‐old girls. ^c^Source: Spiller et al. (14). Self‐poisoning among 13‐ to 15‐year‐old girls. ^d^Source: Twenge et al. (11). Major depressive episode among 14‐ to 15‐year‐old girls. ^e^Sources: Keyes et al. (8) and Twenge et al. (9). Depressive symptoms among eighth‐grade girls.

These mental health indicators have shown similar patterns, with increases beginning in the early 2010s (Figure 1), when iGen/GenZ (born 1995–2012) began entering adolescence, succeeding millennials (born 1980–1994) (17). In most cases, the increases in indicators of poor mental health have been larger among girls and young women than among boys and young men. Many of these indicators have increased considerably: self‐poisonings among 10‐ to 12‐year‐old girls quadrupled (14); hospital admissions for self‐harm tripled among 10‐ to 14‐year‐old girls (12); major depressive episode among 12‐ to 17‐year‐old girls increased 52%, from 13.1% in 2005 to 19.9% in 2017 (11); emergency room visits for suicidal ideation and attempts nearly doubled among children and adolescents (15); and suicide among 10‐ to 14‐year‐old girls doubled (16).

Thus, after improvement or stability during the early 2000s, U.S. adolescents’ mental health worsened considerably during the 2010s. Because these trends included increases in objectively measured behaviors linked to mental health issues (self‐harm, suicide attempts, and suicides), they cannot be dismissed as stemming from greater help‐seeking, awareness of mental health conditions, or willingness to admit problems. Similar increases in mental health issues have also appeared among adolescents in the United Kingdom and Canada (Haidt and Twenge, 2019, unpublished). With such similar trends across many measures and sources, an overwhelming amount of evidence indicates that adolescent mental health has deteriorated since 2010.

The more difficult question to answer is why this trend has occurred. It seems unlikely that these trends have been caused by economic factors, given that the U.S. economy improved from 2012 to 2018. Even if some of the distress originated in economic troubles, it seems unlikely that it would peak from 2017 to 2018, when the economy was strongest. In addition, the largest increases in mental health problems have appeared among the youngest teens, the age group least likely to be concerned with economic issues. Other economic trends, such as income inequality and the decline of the manufacturing sector, have been ongoing since the 1980s and did not suddenly increase after 2012 (18).

There is a growing consensus that the decline in mental health may be linked instead to the increasing popularity of smartphones and social media around this same time (8, 9, 14, 19). For example, smartphone ownership exceeded 50% by the end of 2012 and reached 81% by 2018 (20). Similarly, daily use of social media increased among teens, from about 50% to more than 80%, and time spent online doubled (21). Unlike the economic recession, the effects of which have receded, the impact of increased technology use has continued to grow. Although digital media use among adolescents has benefits for connections and friendships, there also may be costs, especially for excessive use (22).

## Possible Mechanisms by Which Digital Media Use May Influence Mental Health Trends

If the sharp increase in mental health issues is connected to the rise of digital media and electronic device use, it is important to understand how such use may contribute to mental health. The simplest explanation is that more frequent digital media use leads to mental health issues, so as digital media use rises, mental health issues will also rise. However, this explanation does not determine why more frequent digital media use may cause mental health issues, nor does it explore consequences of digital media use beyond time spent. In addition, it does not recognize that effects operate not just at the individual level but also at the group level, such that the rise in digital media use among adolescents may affect even those who are not frequent users.

Below, I explore six mechanisms by which digital media use may be related to mental health and happiness. I define digital media use as including social media, online game playing, texting, other online activities, and streaming videos. Given the focus of studies to date, digital media use in this review refers to use during leisure time and not to use for work, school, or homework.

### Displacement of In‐Person Social Interaction (Individual Level)

Studies of large, representative samples of adolescents (22, 23, 24, 25, 26, 27, 28) have found that heavy users of digital media are more likely to be unhappy, depressed, or have risk factors for suicide (29). In many cases, however, the literature shows that nonusers of technology are slightly worse off than light users (those who use technology about 1 hour/day), with well‐being declining with progressively higher levels of use in an exposure‐response pattern. This finding suggests that heavy use, not digital media itself, is the issue. Heavy digital media use may displace time that might otherwise have been spent in in‐person social interaction, an activity with established links to better mental health and happiness (21, 30).

**FIGURE 2:**
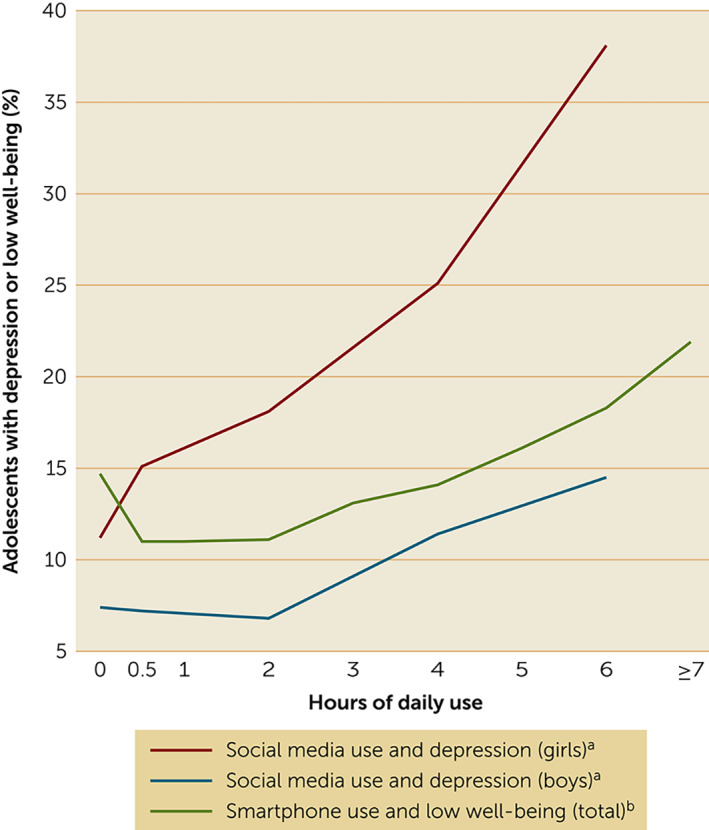
Proportion of adolescents with depression or low psychological well‐being, by hours a day of social media or smartphone use ^a^Source: Kelly et al. (23). ^b^Source: Przybylski and Weinstein (25) and reanalyzed by Twenge and Campbell (28).

Although some studies have concluded that the associations between digital media use and well‐being are small (25, 31, 32), several of these studies included television use in their calculations of effect size. The association between television watching and well‐being is weaker than the association between digital media use and well‐being. Furthermore, because television watching among teens has declined since 2010 (21), this activity cannot credibly explain the increase in teen depression. In addition, several studies controlled for variables (e.g., physical activity or attitudes toward school) that may mediate the associations between technology use and well‐being; this approach can lower effect sizes by explaining away mechanisms of interest and is thus discouraged in correlational modeling (33). Furthermore, most of the studies that have shown small effects used percentage of variance explained (r‐squared), an analysis technique that can minimize and distort effects (34). For example, r=0.20 is twice that of r=0.10, but, after squaring, 4% is four times as large as 1%, leading Funder and Ozer (34) to describe the r‐squared approach as misleading. When other analysis techniques have been used, effect sizes for the same data sets have been considerable (Figure 2). For example, Kelly et al. (23) found that twice as many heavy users of social media (versus nonusers) had clinical levels of depressive symptoms in the Millennium Cohort Study, the same data set that Orben and Przybylski studied (32). Similarly, Twenge and Campbell (28), with the same data set studied by Przybylski and Weinstein (25), found that heavy users of smartphones were twice as likely as light users to experience low well‐being.

Przybylski and Weinstein (25) and Orben and Przybylski (32) noted that the associations they identified between well‐being and screen time were smaller than those for other common risk factors for mental health problems, such as binge drinking, marijuana use, or skipping breakfast. However, they did not mention that, in the same data set, social media use among girls was more strongly associated with depression than was heroin use, exercise, or obesity (35) or that the association was similar to the correlation between childhood lead exposure and IQ (36). With arbitrarily chosen comparison variables, it can be difficult to judge practical importance. It would be helpful if future studies used the same statistical techniques to analyze associations. Because of the curvilinear pattern, with the inflection point often around 1 to 2 hours of screen time per day, a relative risk or odds ratio calculation comparing use of <2 hours and >2 hours a day might be helpful. Another possible method may be to compare heavy users of digital media with light users. Both of these techniques compare the number of people affected, a useful gauge of practical importance (34).

In addition, it is important to note that most causes of mental health issues (e.g., genetic predisposition) do not change in short periods of time. Thus this discussion is not focused on all possible causes of mental health issues, but instead on what changed in the lives of adolescents while mental health issues among adolescents increased. The rapid and widespread adoption of digital media and their impact on the daily lives of adolescents suggest they may play a role in this increase.

Many studies examining associations between digital media use and well‐being have been cross‐sectional and thus unable to determine the direction of causation or to rule out all possible confounding variables. However, two random‐assignment studies on late adolescents and adults found that limiting social media use lowered depressive symptoms (37, 38), and another study found that even 20 minutes of social media use lowered happiness (39). These studies suggest that at least some of the increase in mental health issues has been driven by social media use. More research is needed on the effect of social media on adolescent mental health, especially with larger and more representative samples.

### Displacement of In‐Person Social Interaction (Generational Level)

Displacement is usually understood at the individual level: if an individual spends more time on digital media, she or he has less time to spend on other activities. However, because many activities compete for time, digital media time and in‐person time are not mutually exclusive. For example, likely because of individual differences in sociability, adolescents who spend more time on social media also spend more time with friends in person (7). Their social interaction time, both online and offline, may displace time that might otherwise have been spent on homework, paid work, or other activities.

However, displacement can also take place at the group or generation level, where the focus is on the behavior of the average teen in one year versus another year. For example, among adolescents, as average time spent on digital media rose after 2010, average time spent in person declined (7), even though time spent on paid work, homework, and extracurricular activities declined or stayed steady (40). This finding suggests that digital media time displaced in‐person time at the generation level; the cultural norm for adolescents shifted away from in‐person interaction and toward digital interaction.

This change in cultural norms has implications for mental health across the adolescent population. Even if an individual adolescent chooses not to use digital media or to use it lightly—thus achieving immunity from individual‐level effects on mental health—the prevailing cultural norm may have an impact. Imagine Emma, a 13‐year‐old in 2020 who does not use social media. Social media use cannot increase Emma's depression directly (at the individual level) because her use is zero. At the group level, however, Emma is affected. If Emma would rather see her friends in person instead of communicating via social media (as was the norm in the 1990s and earlier), she may be unsuccessful. Who will she go out with if her friends are all at home on Instagram? Thus, the mental health of many adolescents, not just heavy social media users, may suffer over time as the culture of adolescent social life changes. Future research should examine group‐level effects of changes in social norms (e.g., in‐person versus digital communication) among adolescents.

It is not likely that the displacement of in‐person social interaction at the group level affects all age groups equally. For example, social interactions among adolescents likely have changed more profoundly than those among adults. Adolescent culture centers around peers of similar age, so generational shifts will be more apparent and consistent among adolescents than among middle‐aged and older adults, who are more likely to have relationships with people of varying ages. It is not likely that time spent in in‐person social interaction has changed as much among middle‐aged and older adults as among adolescents, which may be one reason mental health trends since 2012 have been more negative for adolescents and young adults compared with older individuals. Impacts may also differ by gender. Girls spend more time on social media, and boys spend more time playing online games. If social media use is more strongly associated with depression than game playing is and is more strongly associated with depression among girls (23, 41), that may explain why depression rates have increased more sharply among girls. Changes in social interaction may also have more impact on girls than boys. Girls tend to spend more time in friendship dyads whereas boys spend more time in groups (42), and girls focus more on social relationships and popularity than boys do (43), especially during adolescence (44). Girls’ friendships are both more intimate (45) and more fragile (46) than those of boys. Perhaps as a result, adolescent girls’ moods are more influenced by interpersonal events than are boys’ moods (47, 48). Thus, trends in social interaction are likely to have a larger impact on girls’ mental health than on that of boys, and the increases in mental health issues have, in fact, been larger among girls and young women.

### Interference With In‐Person Social Interaction

Unlike previous technological innovations, such as television, smartphones are portable and are often present during social interactions. A series of studies has demonstrated that the presence of a smartphone during social interactions lessens enjoyment of the activity and decreases pleasant interactions among people (49, 50, 51). In addition, several studies have established the negative emotions associated with having one's interaction partners looking at their phones—an action known as phubbing (a combination of “phone” and “snubbing”) (52). Thus, digital media can directly interfere with the benefits of in‐person social interaction when it does occur.

### Interference With Sleep

Time spent on digital media may also displace sleep time. This displacement has been shown at the generational level, where the amount of sleep among adolescents began to fall around 2012 (53), and at the individual level, where those who use digital media more frequently sleep less (54, 55). Randomized controlled trials (56) also show shorter and compromised sleep after digital media use. Digital media use may also increase sleep latency (the time it takes to fall asleep) and sleep quality, because the blue light from devices suppresses melatonin production (57). Shortened and poor quality sleep are known risk factors for mental health issues, especially depression (58). Future research should explore how digital media use affects sleep, especially whether engaging in different digital media activities immediately before bedtime (e.g., social media use versus playing games) has differing effects.

### Cyberbullying and Toxic Environments

Many adolescents experience cyberbullying (i.e., bullying via digital media). As digital media use has become the norm, more adolescents are vulnerable to cyberbullying for more hours. Cyberbullying is a significant risk factor for suicidal ideation and attempts (59) and has been identified as a key mediator for the link between social media use and mental health (60).

Even if online behavior does not reach the level of cyberbullying, online environments can become uncivil more quickly than in‐person environments (61, 62). Digital media gives the impression of anonymity, which can result in an increase in incivility and aggressive behavior known as the online disinhibition effect (63). Social media may also foster unhealthy amounts of social comparison, with users feeling that their lives do not measure up to the lives of others (64, 65). In particular, social media comparison may increase body image concerns, especially among girls (9).

### Self‐Harm Information and Contagion

Although there are many advantages of instant access to information via the Internet, that accessibility also means that adolescents can easily find information about self‐harm and suicide techniques (for example, via YouTube) (66). With more children and younger adolescents having their own smartphones, more may have access to information about self‐harm. This access may fuel contagion behaviors, with adolescents encouraging each other to self‐harm and self‐harm appearing popular (14). This mechanism may operate relatively independently from the amount of time spent on digital media, because children and adolescents may access self‐harm information quickly. Thus, the connection between digital media and self‐harm may be more about the specific activities engaged in online, rather than about how much time adolescents spend online.

Practitioners may need to consider digital media use when counseling adolescents who have mental health issues, particularly depression and self‐harm behavior. Suggestions such as keeping devices out of the bedroom at night, not using devices within an hour of sleep time, and limiting digital media use during leisure time to ≤2 hours a day may be warranted. In most cases, limiting (rather than eliminating) device use may be the best course, but in more acute cases (such as self‐harm contagion) elimination of device use for a period of time may be necessary. More research is needed to explore therapy and treatment techniques for situations in which digital media use may be contributing to mental health issues, and randomized controlled trials are needed to explore the effectiveness of the above suggestions.

## Conclusions

In conclusion, we are in the midst of a mental health crisis among adolescents in the United States. This crisis will necessitate more resources for treatment in the coming years and potentially far into the future, given that those who experience their first episode of depression as adolescents are more likely to relapse as adults (67). The cause of this crisis is difficult to determine, but the rise of digital media is one possibility. The interaction between digital media use and mental health is complex, appearing to operate directly and indirectly, at the individual and generational levels, and via time spent as well as through specific experiences. With more adolescents diagnosed as having depression and engaging in self‐harm behavior and suicide attempts, it is imperative to not only treat those with these conditions but to explore how to prevent their onset.
